# Determination of Pesticide Residues in Honeybees using Modified QUEChERS Sample Work-Up and Liquid Chromatography-Tandem Mass Spectrometry

**DOI:** 10.3390/molecules19032911

**Published:** 2014-03-06

**Authors:** Żaneta Bargańska, Marek Ślebioda, Jacek Namieśnik

**Affiliations:** 1 Department of Analytical Chemistry, Faculty of Chemistry, Gdansk University of Technology, Narutowicza 11/12, Gdańsk 80-233, Poland; E-Mail: jacek.namiesnik@pg.gda.pl; 2 Technologies Polska Sp. z.o.o., Puławska 303, Warszawa 02-785, Poland; E-Mail: mslebioda@perlan.com.pl

**Keywords:** environmental monitoring, honey, honeybees, LC-MS/MS, pesticides, QuEChERS method

## Abstract

Increasing emissions of chemical compounds to the environment, especially of pesticides, is one of factors that may explain present honeybee colony losses. In this work, an analytical method employing liquid chromatography-tandem mass spectrometry (LC-MS/MS) was optimized for the simultaneous screening of 19 pesticides which have not been yet determined in honeybee samples from northern Poland (Pomerania). The sample preparation, based on the QuEChERS method combining salting-out liquid-liquid extraction to acetonitrile and a dispersive-SPE clean-up, was adjusted to honeybee samples by adding a small amount of hexane to eliminate beeswax. The recovery of analytes ranged from 70% to 120% with relative standard deviation ≤20%. The limits of detection were in the range of 0.91–25 ng/g. A total of 19 samples of honeybees from suspected pesticide poisoning incidents were analyzed, in which 19 different pesticides were determined.

## 1. Introduction

Nowadays, pesticides are widely used in agricultural practice to control pests and diseases. Degradation of these compounds in the environment and extensive or inappropriate use by farmers can lead to the contamination of various ecosystems. Widespread distribution of pesticides is also known to cause problems to the apiculture industry. Bees may be contaminated by pesticide residues during harvesting and contaminants can be transported on bee bodies or with forages to the hive, from where they can be transferred into honey. Moreover, the use of pesticides in beehive treatment (during honey harvesting) is another possible route of honey contamination. The presence of such xenobiotics in bee products can decrease their quality and devalue their properties [1,2,3,4,5,6].

Honeybees can be used as indicators of environmental pollution because of their morphological characteristics and the intense foraging activity, and their ability to retain and bioaccumulate in their bodies substances which they are in close contact with during pollination [7,8,9,10].

Increased mortality of bee colonies was observed in the USA in 2006 [11]. Most honey bee and bumble bee losses are partly attributable to pesticide exposure [12,13] and some European beekeepers have reported major losses of honey bee colonies located near crops treated with pesticides, even at a low dose [14]. The phenomenon of mass extinction of bees is called Colony Collapse Disorder (CCD) [15,16]. It should be noticed that also parasitic flies, mites, nutritional stress and decreased biodiversity caused by industrial agriculture may be other important factors conditioning the occurrence of Colony Collapse Disorder [17,18,19,20,21].

Chronic exposure of honeybees to pesticides at concentrations that could approximate field-level exposure impairs their natural foraging behavior and increases worker mortality leading to significant reductions in brood development and colony success [22]. Consequently, determination of residues of these compounds is very important. Determination of pesticides in honeybees at trace levels is a challenging task due to the complex sample matrix. Honeybees contain a large amount of beeswax, proteins and other substances which have an adverse effect on the results of analyses. Therefore, clean-up stage prior to analysis is often necessary. Matrix solid-phase dispersion (MSPD) [23,24,25] or solid phase extraction (SPE) followed by clean-up using gel permeation chromatography (GPC) [26,27] is the most frequently utilized for honeybee sample preparation stage. However, this procedure of preparing samples for analysis allows only for the determination of selected analytes from the group of pesticides. New ways of sample preparation for analysis are necessary to determine the widest possible spectrum of pesticides.

The multiresidue methods have become more popular in recent years because they can be used for the determination of a wide range of compounds in one analytical process. The most universal extraction technique to isolate a wide range of pesticides is the QuEChERS method, first introduced in 2003 [28]. This method ensures excellent extract clean-up and high analytes recovery [29] in application to different food matrices such as fruits and vegetables [30,31,32,33,34,35], fruit juices [36], raisings and wheat flour [37], cereals and fish tissue [38], rice paddies [39], soil [40], olives and olive oil [41], milk, eggs, avocado [42], honey [43,44] and honeybees [44,45,46]. 

In this article, we describe the evaluation and adaptation of the QuEChERS approach in combination with liquid chromatography–tandem quadrupole mass spectrometry (LC–MS/MS) used to determine 19 pesticide residues in honeybee samples. The developed methodology was already used to determine other types of pesticide residues in different matrices (honey samples) [47]. Based on the previously obtained data, samples of dead honeybees were collected from the most contaminated areas of Pomerania in Poland (Tczew, Gdansk, Kartuzy) where pesticide poisoning was suspected. The methodology was optimized and next it was validated according to the regulation presented in the Method Validation and Quality Control Procedures for Pesticide Residues Analysis in Food and Feed [48]. This methodology allows for the monitoring of pesticides belonging to various classes for the use of bee organisms as the indicators of environmental contamination.

## 2. Results and Discussion

### 2.1. Extraction Procedure

Bee samples always contain large amounts of wax, proteins and other substances readily extractable with organic solvents [40]. The separation of co-extracted beeswax from extract samples containing the pesticide residues of interest was the main challenge in developing the clean-up method. The basic QuEChERS method (using 10 mL of water and 10 mL of acetonitrile with a set of packaged salts precipitation) does not eliminate beeswax, which was observed in the resulting extract. A modification by the freezing of the extract before the purification with dSPE (2 h, −24 °C) was introduced, but a reduction of recovery of the analytes was observed. Therefore, during further optimization of the extraction stage, 3 mL of *n*-hexane was added before application a set of packaged salts followed by dSPE purification.

**Figure 1:**
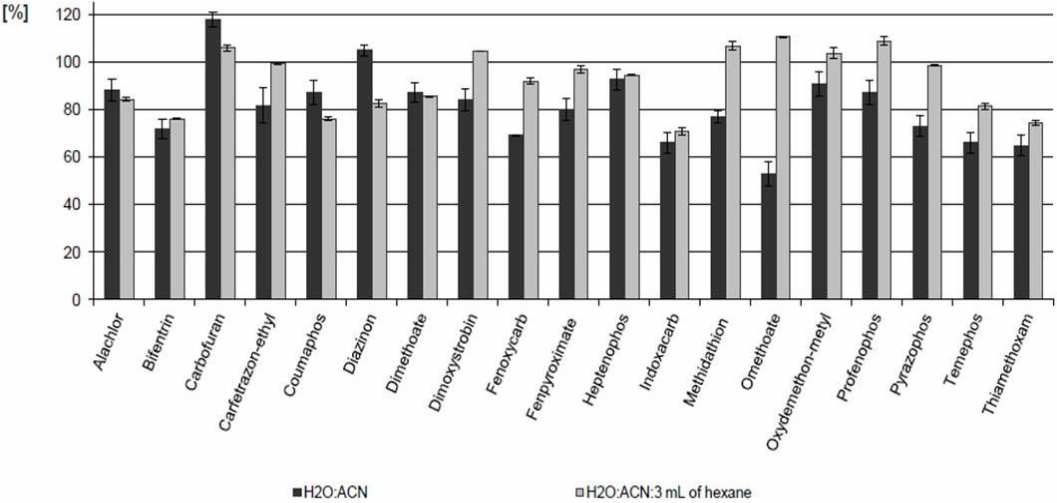
Recovery changes during the optimization of the extraction process.

The addition of 3 mL of n-hexane helped to remove the interfering matrix components, especially wax. Figure 1 presents differences in the recovery found during the optimization of the extraction process. The recovery of some analytes was lowered by the addition of *n*-hexane to a small extent (4% for alachlor and dimethoate, 12% for carbofuran and coumaphos). A greater difference (22%) was observed for diazinon. For other pesticides, better recoveries were achieved by applying a mixture of water/acetonitrile/hexane in the extraction process in comparison with the mixture of water and acetonitrile.

### 2.2. Method Performance

**Figure 2:**
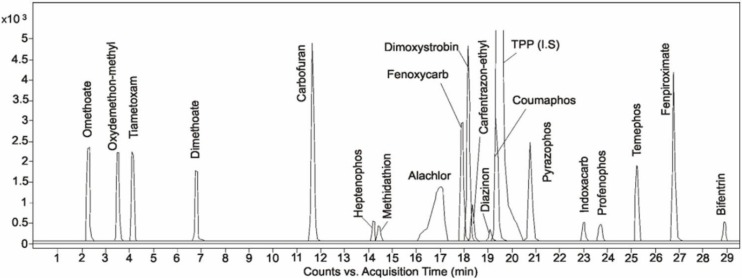
Sample MRM chromatograms of honeybee sample spiked with pesticides at concentration level of 3LOQ.

The developed method was evaluated according to the Method Validation and Quality Control Procedures for Pesticides Residues Analysis in Food and Feed [48] in terms of repeatability, linearity, recovery and precision. The selectivity of the method was assessed by analysis of honeybee samples and spiked honeybee samples with addition of TPP 100 ng/g (I.S). No significant interferences were observed (Figure 2).

The limits of detection (LOD) and quantification (LOQ) were estimated based on the preliminary calibration curve in acetonitrile within the concentration range of 2–100 ng/g. The LOD was calculated using the following dependence LOD = 3.3 × SD/b, where b is the slope of calibration curve and SD is the residual standard deviation of the calibration curve. The limit of quantification was calculated as LOQ = 3 × LOD. The appropriate matrix-matched calibration was made at levels of concentrations from 3LOD up to 6LOQ with an addition of 100 ng/g TPP as the internal standard. 

The recovery of the analytes and repeatability studies were performed at two levels of fortification, 3LOD and 3LOQ, by adding known quantities of pesticides to a honeybee sample, each in five replicates (*n* = 5). The mean recovery ranged from 70.1% to 110.6%, as recommended by the SANCO Guideline (Table 1).

**Table 1:** Range of linearity, recovery of analytes, limit of determination and quantification (LOD and LOQ) of the modified analysis method of solvent extracts of honeybee samples.

Analyte	Range [ng/g]	Recovery [%] ± RSD [%] *n* = 5		LOD [ng/g]	LOQ [ng/g]
		LOQ	3LOQ		
Alachlor	75/450	80.5 ± 5.4	84.34 ± 0.76	25	75
Bifentrin	4.05/24.40	85 ± 17	76.08 ± 0.22	1.3	4.1
Carbofuran	3.65/22.00	106 ± 11	106.1 ± 1.2	1.2	3.6
Carfetrazon-ethyl	3.78/22.60	79.0 ± 9.0	99.48 ± 0.45	1.3	3.8
Coumaphos	4.95/29.80	80.4 ± 7.7	76.18 ± 0.95	1.6	4.9
Diazinon	4.02/24.20	83 ± 14	82.7 ± 1.6	1.3	4.0
Dimethoate	3.51/21.00	85.3 ± 7.2	85.63 ± 0.28	1.2	3.5
Dimoxystrobin	3.87/23.20	76.8 ± 5.7	104.45 ± 0.40	1.3	3.9
Fenoxycarb	3.69/22.20	70.8 ± 6.3	91.9 ± 1.3	1.2	3.7
Fenpiroxymate	3.69/22.20	83 ± 10	97.0 ± 1.5	1.2	3.7
Heptenophos	2.97/17.80	93.0 ± 8.0	94.63 ± 0.10	1.0	3.0
Indoxacarb	3.66/22.00	70.8 ± 5.1	70.8 ± 1.6	1.2	3.7
Methidathion	4.53/27.20	74.6 ± 6.7	106.8 ± 1.8	1.5	4.5
Omethoate	3.63/21.80	71.6 ± 2.1	110.58 ± 0.14	1.2	3.6
Oxydemeton-methyl	3.63/21.80	87.3 ± 9.3	103.9 ± 2.2	1.2	3.6
Profenophos	3.42/20.60	80.1 ± 5.7	109.1 ± 1.7	1.1	3.4
Pyrazophos	3.87/23.20	70.4 ± 1.8	98.69 ± 0.32	1.3	3.9
Temephos	3.69/22.20	72.70 ± 0.36	81.5 ± 1.2	1.2	3.7
Thiamethoxam	3.42/20.60	70.1 ± 2.0	74.38 ± 0.92	1.1	3.4

### 2.3. Application to Real Samples

The methodology described above was used to monitor pesticide concentrations in 19 honeybee samples obtained from the Regional Beekeepers Association in Gdansk (Poland) in the year 2013. Detailed information about concentration levels of pesticides residues found in the analysis of honeybee samples is presented in Table 2.

**Table 2:** Pesticide residues determined in honeybee samples collected from apiaries in the Pomerania region of Poland (concentration with expanded uncertainty).

Pesticides	Number of samples	The districts in Pomerania (Poland)	No. of samples above LOD (%)	Min level [ng/g] (RSD)	Max level [ng/g] (RSD)
Alachlor	11–15	Kartuzy, Tczew	5 (26.3%)	>LOD	95.0 (8.4)
Bifenthrin	1, 5, 6, 7, 10–14, 19	Gdansk, Kartuzy, Tczew	10 (52.6%)	nd	<LOQ
Carbofuran	1, 11	Gdańsk, Kartuzy	2 (10.5%)	nd	<LOQ
Carfentrazon-ethyl	1, 11, 14, 15	Gdańsk, Kartuzy, Tczew	4 (21%)	>LOD	18.1 (1.3)
Coumaphos	11	Kartuzy	1 (5.3%)	nd	<LOQ
Diazinon	1, 5, 11–13, 15	Gdańsk, Kartuzy, Tczew	6 (31.6%)	>LOD	13.3 (1.4)
Dimethoate	1, 8, 13, 14, 16	Gdansk, Kartuzy, Tczew	5 (26.3%)	>LOD	20.5 (0.4)
Dimoxystrobin	11	Kartuzy	1 (5.3%)	nd	<LOQ
Fenoxycarb	11	Kartuzy	1 (5.3%)	nd	15.0 (0.2)
Fenpyroximate	1, 11	Gdansk, Kartuzy	2 (10.5%)	nd	<LOQ
Heptenophos	1, 2, 3, 6, 8–16	Gdańsk, Kartuzy, Tczew	13 (68.4%)	>LOD	18.5 (0.6)
Indoxacarb	11, 12	Kartuzy	2 (10.5%)	>LOD	11.8 (2.3)
Methidathion	1, 5, 6, 10, 11, 13–16	Gdańsk, Kartuzy, Tczew	9 (47.4%)	>LOD	22.4 (1.9)
Omethoate	11, 14, 16	Kartuzy, Tczew	3 (15.8%)	>LOD	15.8 (2.9)
Oxydemeton-methyl	11	Kartuzy	1 (5.3%)	nd	18.4 (1.5)
Profenophos	1, 11, 12	Gdańsk, Kartuzy	3 (15.8%)	>LOD	7.6 (2.7)
Pyrazophos	2, 5, 6, 10, 11, 15	Gdańsk, Kartuzy, Tczew	6 (31.6%)	>LOD	14.3 (0.6)
Temephos	1, 2, 13	Gdańsk, Kartuzy	3 (15.8%)	nd	<LOQ
Thiamethoxam	11, 14	Kartuzy, Tczew	2 (10.5%)	>LOD	4.1 (0.7)

nd—not detected.

The investigated pesticide residues were detected in all 19 honeybee samples. Heptenophos (organophosphorus insecticide) was detected in 68.4% of the samples. Laboratory tests have shown that it is highly toxic to bees and can be accumulated in bee products [49]. Bifenthrin was detected in 52.6% of samples. This is an insecticide and a member of the pyrethroid family of chemicals. Bifenthrin is very highly toxic to bees (neurotoxic, typically causing paralysis in target pests) with a reported oral LD_50_ of 0.1 μg/bee and contact LD_50_ of 0.01462 μg/bee (about 1,000 ng/g and 150 ng/g, respectively) [50,51].

Methidathion was detected in 47.4% of the samples. This pesticide is a non-systemic organophosphorous insecticide and acaricide with stomach and contact action (LD_50_ 0.23 µg/bee). The compound is used to control a variety of insects and mites in many crops such as fruits, vegetables, tobacco, alfalfa and sunflowers. It is also used in greenhouses and on rose cultures. It is especially useful against scale insects. It works by inhibiting certain enzyme actions in the target pests [52]. Methidathion must not be used during the flowering period and bee activity and it is prohibited in the European Union. 

The estimated limit of detection and quantification (25 and 75 ng/g respectively) of alachlor is high but alachlor was detected in five samples at levels between the LOQ and 95 ng/g. High levels of this compound in honeybees may be associated with agricultural or apicultural practices in the regions by farmers performing sprayed treatments on the weeds around the apiary without observing the period of prevention. Alachlor is an herbicide used to control annual grasses and broadleaf weeds in field corn, soybeans, and peanuts. It is a selective systemic herbicide, absorbed by germinating shoots and by roots. It works by interfering with a plant’s ability to produce protein and by interfering with root elongation [53].

One of the samples (number 11, Kartuzy district) was contaminated by 17 of the 19 pesticides under analysis. On the basis of an analysis of the results obtained it can be concluded that the accumulated contamination, such as pesticides in bee organisms collected from the investigated areas could affect the health of bees and cause their death.

## 3. Experimental

### 3.1. Materials

The solution of triphenyl phosphate (TPP), analytical grade, used as an internal standard was purchased from Sigma Aldrich (Seelze, Germany). The Certified Reference Materials (CRMs) standard solutions of carfentrazon-ethyl, bifenthrin, coumaphos, diazinon, dimethoathe, heptenophos, oxydemethon-methyl, profenophos, pyrazophos, temephos were purchased from LGC Standards (Łomianki, Poland), The CRM solution of alachlor, indoxacarb, carbofuran, fenoxycarb, fenpyroximate, methidathion, omethoate, thiamethoxam was obtained from Ultra Scientific (North Kingston, RI, USA) and CRM solutions of dimoxystrobin were purchased from Dr Ehrenstorfer GmBH (Augsburg, Germany). The stock standard solutions were stored at −18 °C. The calibration standards and working standards were prepared by dilution with acetonitrile on the day of analysis.

Acetonitrile, methanol (LC-MS Chromosolv^®^, ≥99.9%), and n-hexane (pro analysis) were obtained from Fluka (Sigma–Aldrich, Seelze, Germany). Acetic acid and aqueous ammonia were delivered by POCh (Gliwice, Poland). Water was purified with a Milli-Q water system (Millipore Corporation, Billerica, MA, USA). The QuEChERS kits with salt packets containing 4 g of anhydrous MgSO_4_ and 1 g of sodium chloride, as well as two-milliliter centrifuge tubes with 150 mg anhydrous magnesium sulphate and 25 mg primary-secondary amine (PSA) for dispersive solid phase extraction (dSPE) were purchased from Agilent Technologies (Santa Clara, CA, USA). Anhydrous magnesium sulfate and sodium chloride were from Fluka (Sigma–Aldrich), and formic acid was delivered by POCh.

### 3.2. Sample Collection

**Figure 3:**
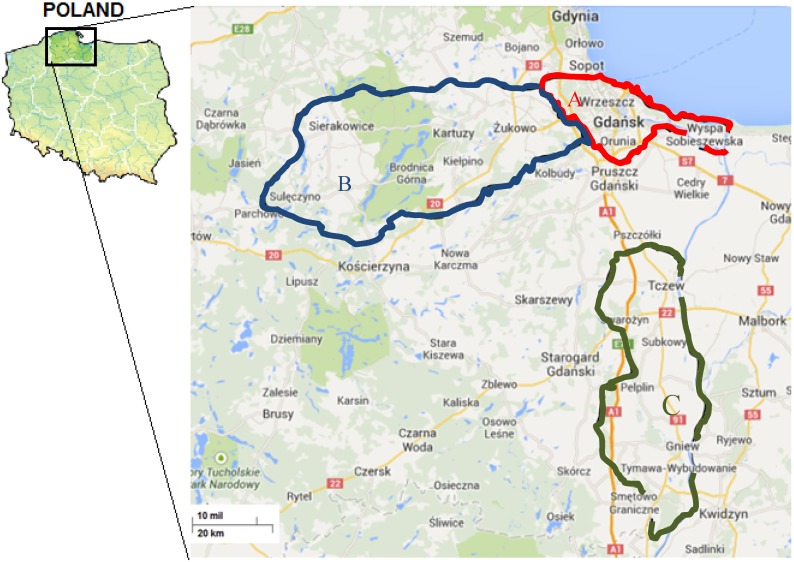
Location of the sample collection area in the northern part of Poland (Pomerania): **A** (Gdansk)—samples 1–7; **B** (Kartuzy)—samples 8–13; **C** (Tczew)—samples 14–19.

The honeybee samples collected in 2013 (April, May, June, July) were submitted to the laboratory by the Regional Beekeepers Association (Gdansk, Poland). All samples were immediately freeze-dried and stored at −18 °C until analysis. Figure 3 presents the location of the sample collection area in the northern part of Poland (The Pomerania Voivodeship or The Pomerania).

### 3.3. Sample Preparation

**Figure 4:**
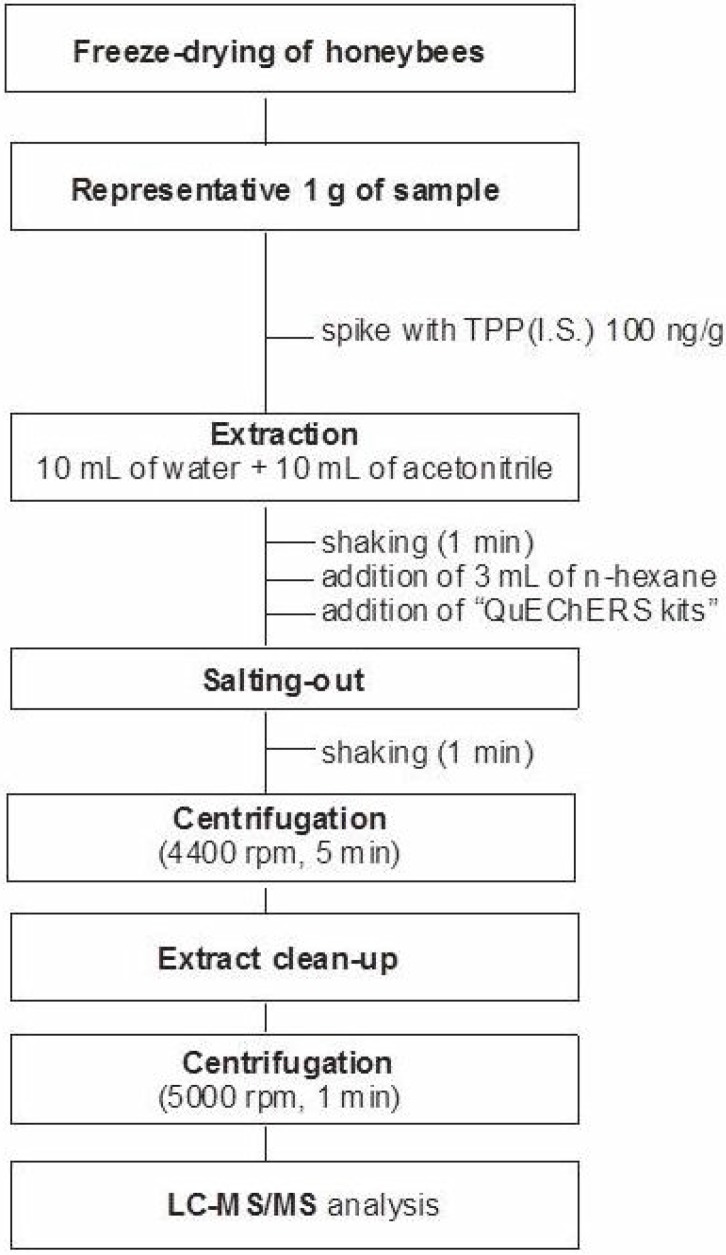
The procedure of modified QuECheRS/d-SPE sample work-up for the determination of pesticides in honeybee organisms.

The laboratory samples of freeze-dried honeybees were thoroughly homogenized. Approximately 1 g of sample was weighed into a polypropylene centrifuge tube (50 mL) and acetonitrile (ACN, 10 mL), water (10 mL), *n*-hexane (3 mL), and internal standard solution (TPP at 100 µg/mL, 50 µL) were added. The tube content was hand-shaken. Subsequently, the content of the salt kit QuEChERS was added. The mixture was immediately hand-shaken for 1 min and centrifuged at 4,400 rpm for 5 min. Afterwards, 1 mL of the acetonitrile fraction (below the *n*-hexane fraction) was transferred to a 2 mL dSPE polypropylene tube containing 150 mg of MgSO_4_ and 25 mg of primary secondary amine (PSA). The tube was shaken by hand, vortexed for 1 min and then centrifuged at 5000 rpm for 1 min. Finally, the 0.5 mL aliquot of the supernatant was transferred into a glass autosampler vial. Figure 4 presents the flowchart of all steps of the analytical protocol used during the study.

### 3.4. LC–MS/MS Analysis

An Agilent 1290 Series chromatograph coupled to a model 6460A triple quadrupole mass spectrometer (Agilent Technologies) with a JetStream electrospray source in the positive ionization mode was used. The chromatographic separation was performed on a Poroshell 120 EC-C18 2.7 µm, 3 × 100 mm column (Agilent Technologies). The mobile phase consisting of: (A) water; and (B) methanol, both containing 10 mM of ammonium acetate, was used at a flow rate of 0.4 mL/min. During the analysis, a multi-linear gradient was used from 20% to 50% B in 10 min, 50% to 70% B at 13.5 min, 70% to 71% B at 20 min, 71% to 100% B at 29 min, and 100% B until 35 min. The injection volume of the extract sample was 2 µL.

The capillary voltage was set at 3.5 kV and the electrospray source sheath gas flow and temperature were 5 L/min and 300 °C, respectively. Drying gas was operated at a flow of 11 L/min and a temperature of 250 °C. The nebulizer pressure was maintained at 45 psi. The mass spectrometer was operated in the MS/MS mode using dynamic multiple reaction monitoring (dMRM).

The compounds were identified by their retention times and relative intensities of qualifier ions in the positive ionization mode as listed in Table 3.

**Table 3:** Multiple reaction monitoring parameters of the studied compounds (dMRM: delta retention time 1 min, except for alachlor 2 min).

Compound	MW [g/mol]	Precursor ions (*m/z*)	Product ions (m/z)		t_r_ [min]	Fragmentor potential [V]	Collision energy (CE) [V]
			Quantifier ion	Qualifier ion			
Alachlor	269.1	270.1	238.0	162.1	17.01	103	13
				117.0			61
Bifentrin	422.1	440.2	181.0	165.0	28.94	103	77
				115.0			141
Carbofuran	221.1	222.1	123.0	165.0	11.63	103	9
				51.0			69
Carfetrazon-ethyl	411.1	412.1	345.9	383.9	18.42	152	9
				365.9			13
Coumaphos	362.0	363.0	226.9	334.9	19.43	152	9
				306.9			13
Diazinon	304.1	305.1	169.1	153.1	19.19	103	17
				96.9			33
Dimethoate	229.0	230.0	124.9	198.9	6.70	54	5
				170.9			9
Dimoxystrobin	326.2	327.2	205.0	116.0	18.24	103	17
				58.1			29
Fenoxycarb	301.1	302.1	88.0	256.0	17.98	103	9
				116.0			9
Fenpyroximate	421.2	422.2	366.0	107.0	26.84	152	61
				77.0			101
Heptenophos	250.0	251.0	127.0	125.0	14.17	103	9
				89.0			33
Indoxacarb	527.1	528.1	149.9	292.9	23.13	152	9
				248.9			9
Methidathion	302.0	303.0	145	85.1	14.42	54	13
				58.1			29
Omethoate	213.0	214.0	124.9	182.9	2.19	103	9
				154.9			9
Oxydemethon-methyl	246.0	247.0	168.9	124.9	3.43	103	17
				109.0			25
Profenophos	372.0	373.0	302.8	344.8	23.79	103	9
				128.0			49
Pyrazophos	373.1	374.1	222.0	238.0	20.90	152	17
				148.0			53
Temephos	466.0	467.0	124.9	418.9	25.31	152	13
				404.9			9
Thiamethoxam	291.0	292.0	211.0	181.0	4.05	103	17
				131.9			17

## 4. Conclusions

Pesticides or their residues are persistent and can accumulate in various ecosystems. Honeybees visiting flowers come into contact with pesticides applied to protect crops and ultimately the honey and other bee products become contaminated. The developed analytical procedure allows for determination of 19 pesticide residues in honeybee samples in a single analytical run. The modified sample work-up procedure based on the QuEChERS methodology is effective, economical and fast. The method was applied to determine pesticide levels in real samples from the northern part of Poland (The Pomerania Voivodeship). The results obtained confirm that the death of honeybees occurred mainly as a result of poisoning with pesticide residues remaining near the apiaries and those bees can be used as potential environmental bioindicator.
